# Late embolisation of an atrial septal occluder device in the abdominal aorta

**DOI:** 10.21542/gcsp.2020.37

**Published:** 2020-12-31

**Authors:** Emre Özdemir, Cem Nazlı

**Affiliations:** 1 Izmir Katip Çelebi University, Atatürk Training and Research Hospital, Department of Cardiology, Izmir, Turkey

## Abstract

Percutaneous closure of secundum atrial septal defect (sASD) - which is the most common in adult congenital heart disease - is considered to be the first treatment option but can involve early and late complications. We report on the late embolization of a device to the abdominal aorta, 12 months after successful percutaneous closure of sASD. A 63-year-old woman, who suffered from stomach ache, was found to have an ASD occluder device in her abdominal aorta. Although surgical intervention to remove the embolisation may be considered, medical follow-up and re-intervention of percutaneous closure may be feasible for inappropriate cases.

## Introduction

In the adult population, more than 30% of congenital cardiac defects are atrial septal defects (ASDs) and over 75% of these are secundum ASD (sASD)^1^. Percutaneous closure of secundum atrial septal defects is the preferred treatment, instead of surgery. This method generally proves to be well-tolerated, less painful, with quicker recovery times, and results in shorter hospital stays^2^. However, there are well-known complications which can occur, including cardiac perforations, device embolisation, residual shunts, vascular trauma, atrioventricular valve regurgitation, atrial arrhythmias, and infectious endocarditis^3^.

## Case Report

A 63-year-old female, who suffered from stomach ache, was referred because of a failure to detect an Amplatzer^TM^ Septal Occluder Device (Abbott Medical, Abbott Park, Illinois, U.S.A.) on transthoracic echocardiography 12 months after percutaneous closure of a ASD.

**Figure 1.**
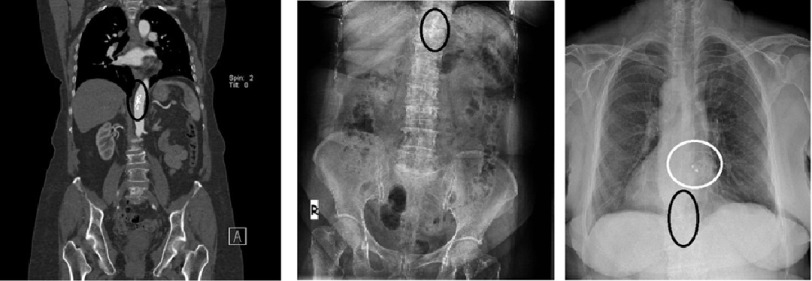
Embolized occluder device is marked in a black ring. Computed tomographic angiography (A) - also can be detected on X-ray before re-intervention (B). New and old-embolized devices can be detected on X-ray (C).

Computerized tomography revealed a 22 mm Amplatzer^TM^ Septal Occluder Device in the abdominal aorta (Figure 1). It was decided not to intervene to remove the device since it appeared to be adhered to the wall of the abdominal aorta. Rather, the patient’s ASD was closed again percutaneously. The sASD size was 23 mm as measured with balloon sizing, and percutaneous ASD closure process was performed successfully with a 26 mm Amplatzer^TM^ Septal Occluder Device. After discharge, patient was followed-up for a period of 3 years, during which she had no complications (Video 1).

## Discussion

Although, there are advances in ASD occlusion techniques and technology, there are also known cases of occluder device complications. The complications of this procedure are cardiac perforations, device malposition or embolisation, residual shunts, vascular trauma, thrombus formation, atrioventricular valve deformations, arrhythmias, infectious endocarditis and death. Generally, most device embolisations occur during the peri-procedural time and are usually recognised and dealt with rapidly. However, late complications such as embolisation often go undetected^5^.

**Video 1.**
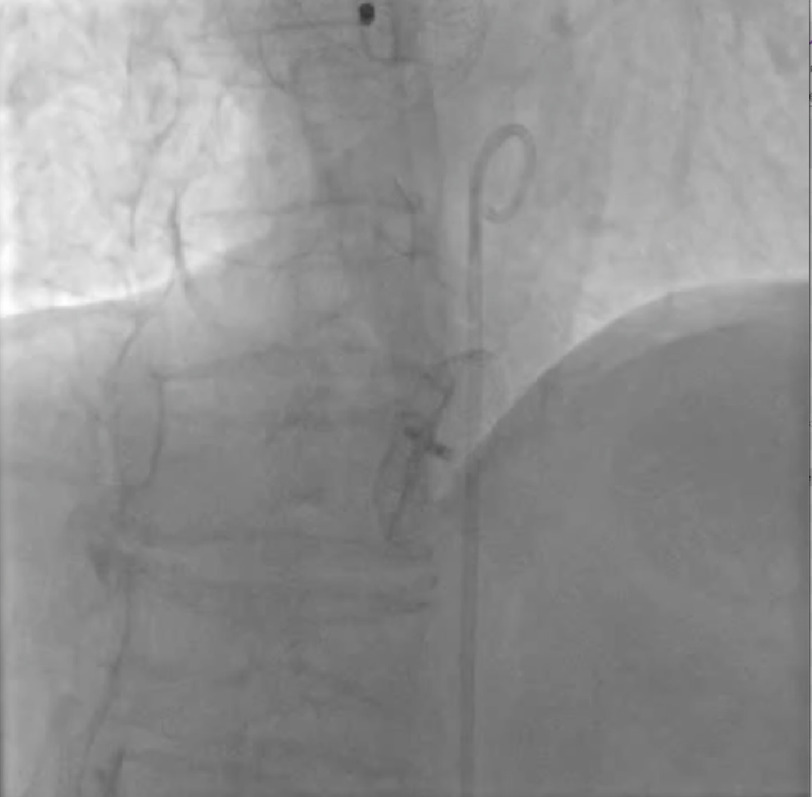
On abdominal aortography, the new device on atrial septum and old-embolized device in the aortic wall, are easily seen. Video available on journal website.

There are a number of reasons for the late failure of percutaneous ASD closure, which include patient and/or device selection failure^6^.

The percutaneous closure of ASD is gaining in popularity. Complications are rare but may be important. Absence of residual shunts and late thromboembolic events may necessitate surgical closure of ASDs^7^. Embolised devices can be retracted percutaneously, especially when detected early. However, some embolisms which migrate to major vascular structures like our patient, need surgical procedures because of the risk of rupture of the aorta^8^. Alternatively, as in the case reported here, medical follow-up can be a solution instead of surgery.

## Conclusion

In our case, surgery to remove embolised device the was not considered. The ASD was closed again with a suitable larger device. Double anti-aggregation treatment was administered for 3 months, and a single-anti-aggregation regime was then continued up to 12 months.
